# Supplementary material for: Genome-scale RNA interference profiling of Trypanosoma brucei cell cycle progression defects
Source: Nat Commun. 2022 Sep 10;13:5326. doi: 10.1038/s41467-022-33109-y (PMC9464253; doi:10.1038/s41467-022-33109-y)
Supplement: Supplementary file 1 — Supplementary Information [file 41467_2022_33109_MOESM1_ESM.pdf]

Genome-scale RNA interference profiling of *Trypanosoma brucei* cell cycle progression defects

Catarina A. Marques, Melanie Ridgway, Michele Tinti,
Andrew Cassidy, David Horn

Supplementary Figures 1-5

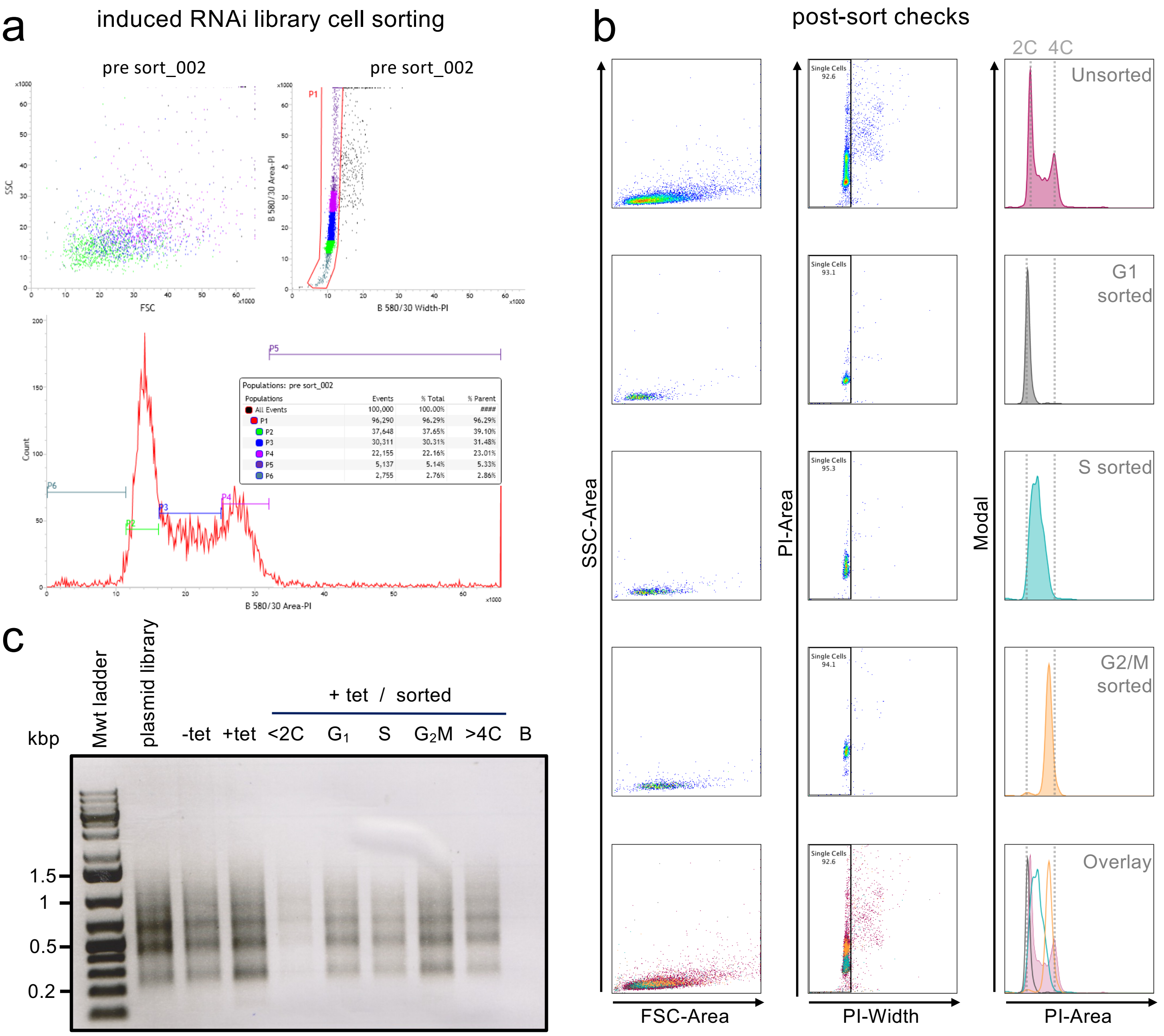

Supplementary Figure 1 Induced library sorting and RNAi target amplification. **a** BD Influx^{TM} cell sorter BD FACSort^{TM} software workspace data for the sorting session showing the gates and population frequencies. **b** Flow cytometry quality control data for the sorted samples. Three types of graphs are shown: SSC-Area x FSC-Area (cell morphology); PI-Area x PI-Width (cells stained with PI; the gate excludes cell aggregates); Modal x PI-Area (cells within the gate set in the PI-Area x PI-Width plot). Top row – unsorted; second row – G_1; third row – S; fourth row – G_2M; bottom row – overlay of the sorted and unsorted samples. 2C and 4C refer to unduplicated (diploid genome) and duplicated DNA content, respectively. **c** PCR amplification of RNAi target fragments from *T. brucei* genomic DNA, using Lib3 primers, and prior to sequencing. B, blank; the plasmid library is also included as a control.

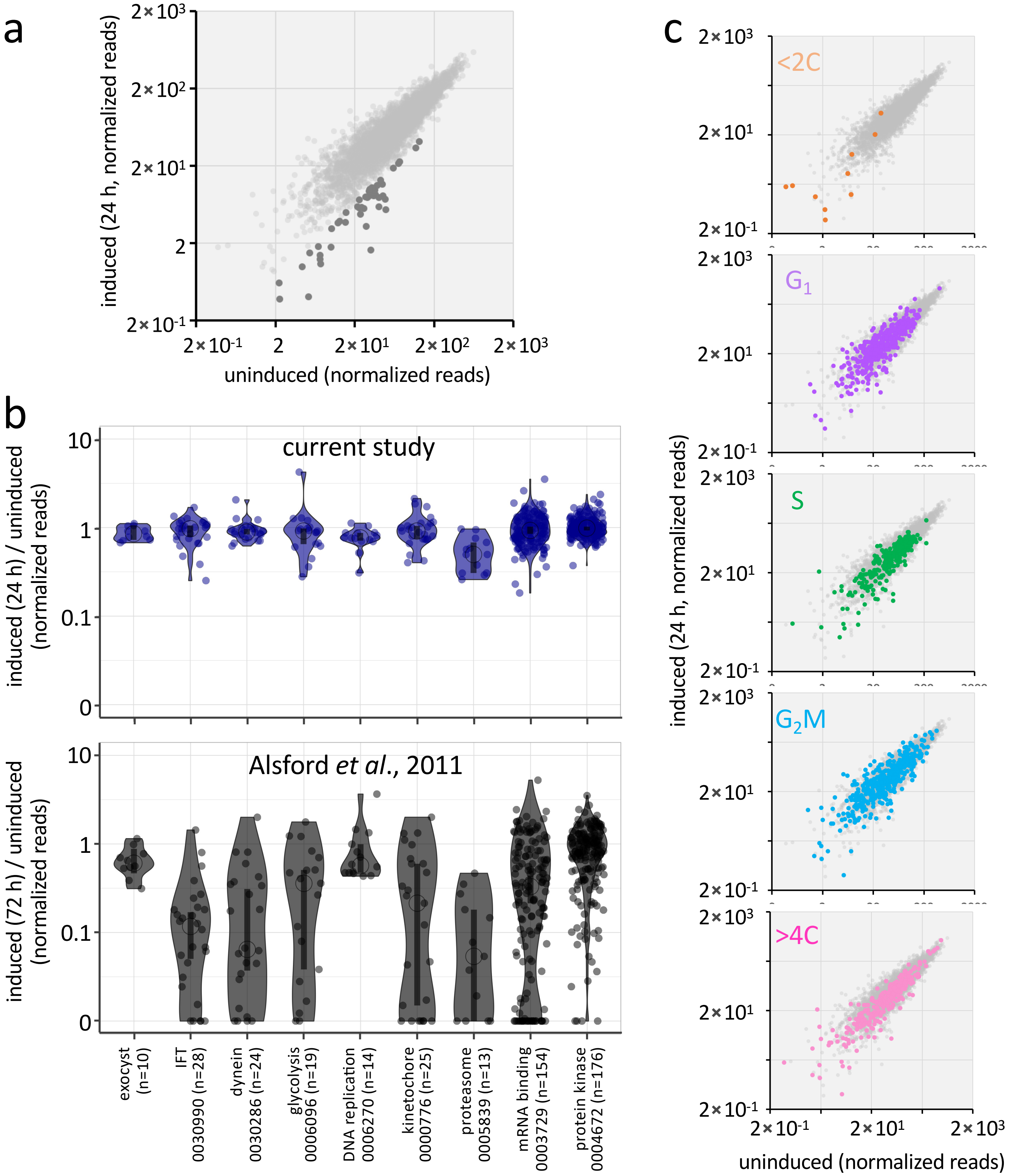

Supplementary Figure 2 RIT-seq data comparing unsorted libraries and showing hit-profiles. **a** The plot shows read-counts for 7,205 genes and for uninduced and 24 h induced samples. Reads for only 0.6% of genes (dark blue) dropped by >3-fold following 24 h of knockdown. **b** The violin plots show relative read-counts for cohorts of genes and reflect data distribution. Open circles indicate median values and the vertical bars indicate 95% confidence intervals. Read-counts remain relatively high after 24 h knockdown in the current study, when compared to read-counts after 72 h knockdown in a prior RIT-seq study. **c** As in a, but showing hits in each of the five arms of the screen (highlighted in each case). Note that several hits in the <2C arm of the screen are under-sampled in terms of read-count.

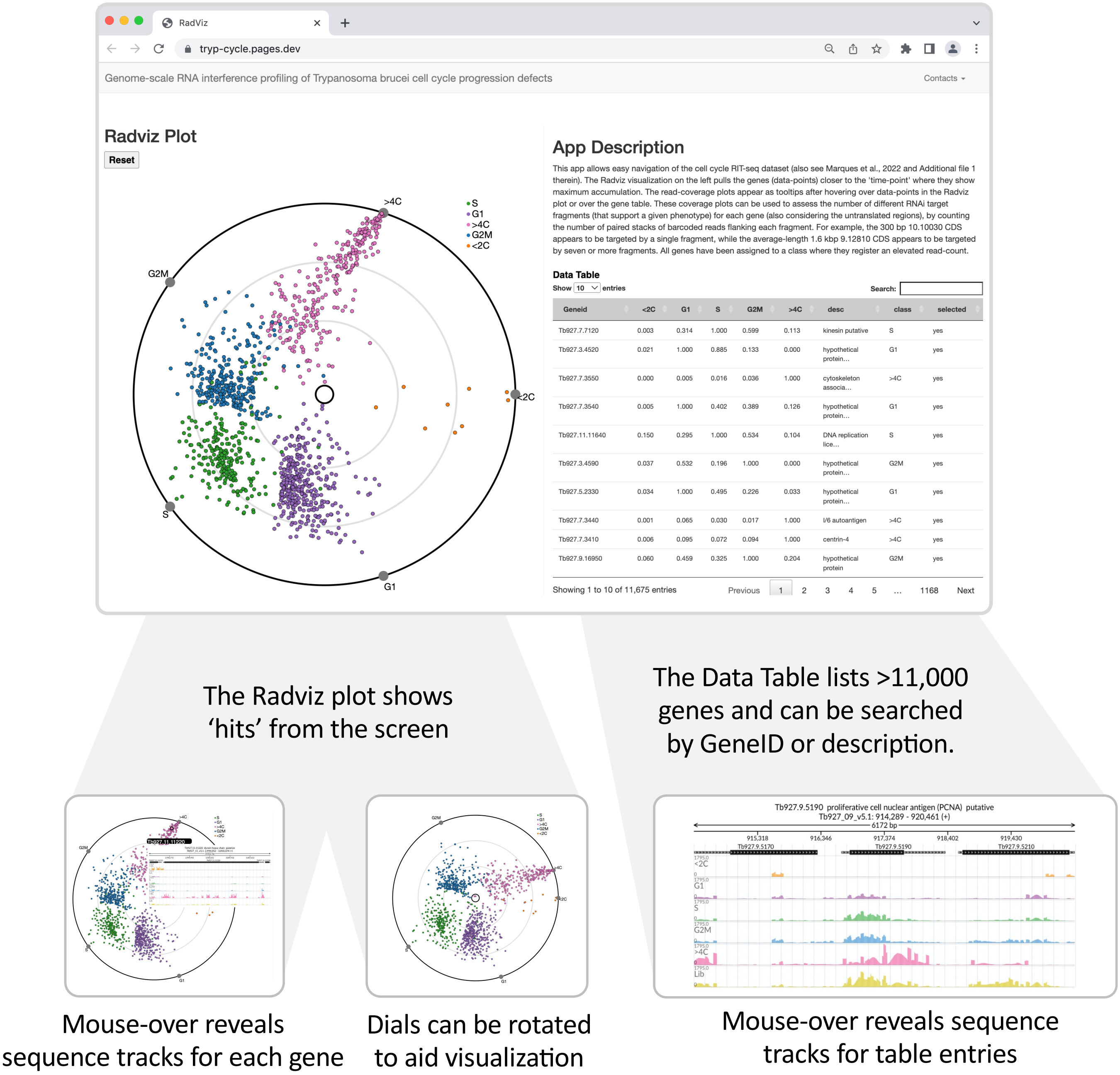

Supplementary Figure 3 Online RIT-seq data visualization (https://tryp-cycle.pages.dev/). In the radial visualization, experimental-points are hours on the clock-face (i.e. related to the angle of the polar coordinate system). The orthogonal axis (i.e. the distance) relates to the relative read-counts across the five experiments. The table on the right shows: Geneid, gene identification number; relative abundance of reads in each sorted sample; desc, gene description; class, the experiment where the gene shows maximum abundance; selected, a binary tag where 'yes' indicates genes in the radial visualization.

Data transformation: Normalised TPM values from Supplementary data 1 were used, except the <2C and >4C values were divided by 7.5 and 2.5, respectively, to maximise alignment with class assignment in Supplementary data 1. To further aid visualization, we elevated the values to the power of 2.1 to maximize differences and, by dividing values by the maximum, normalized the values raw-wise for each gene. The transformed data was then fed to the radial visualization algorithm implemented in D3.js (https://github.com/d3/d3); the code and web page were adapted from the repository at https://github.com/WYanChao/RadViz.

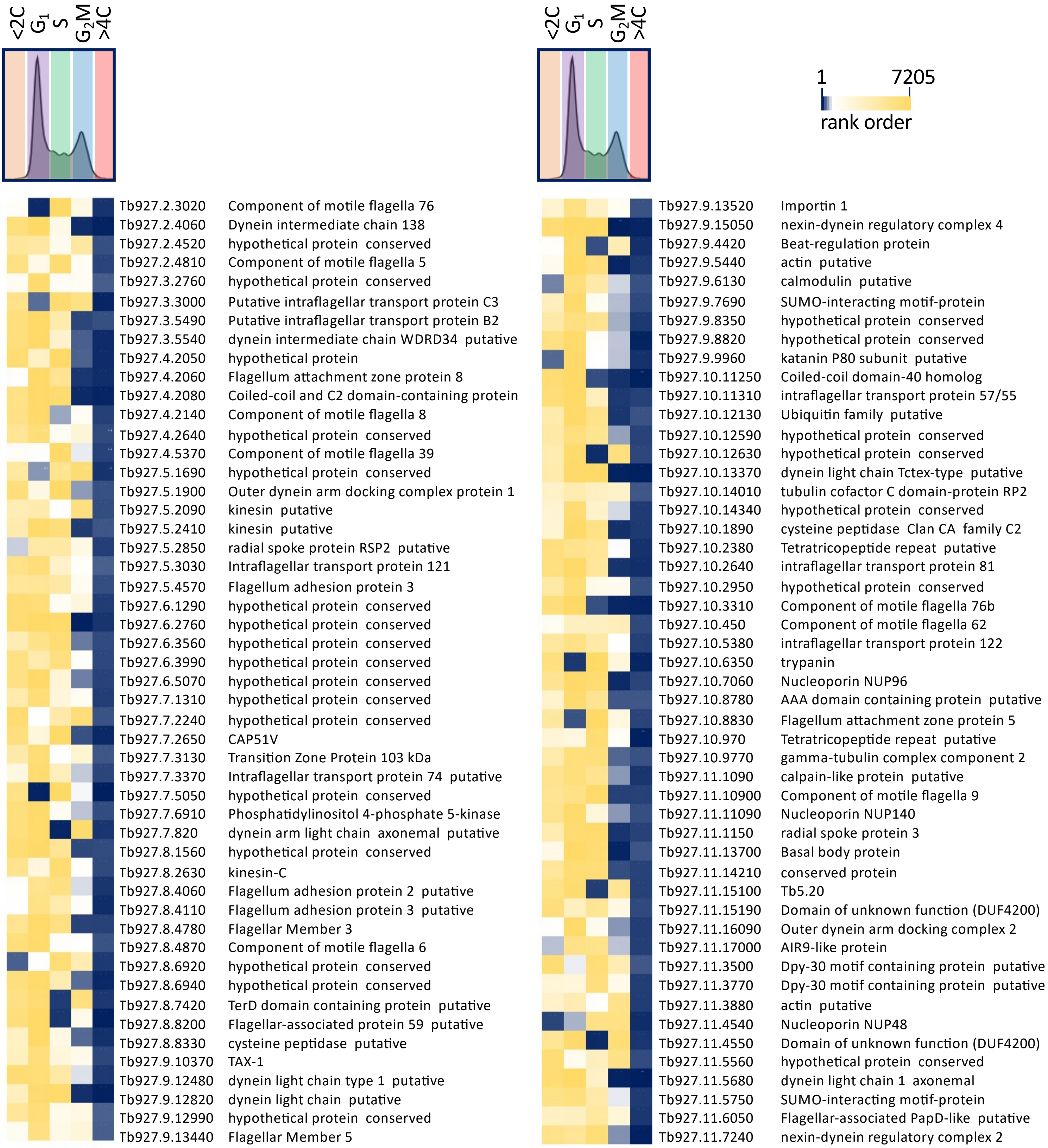

Supplementary Figure 4 Cytokinesis defects associated with endoreduplication. One hundred example RIT-seq cell cycle profiles are shown for hits overrepresented in the >4C pool. Page 1 shows the heatmaps indicating relative representation in all five sorted pools; blue, most overrepresented. Subsequent pages show read-mapping profiles for each gene; see Figure 1b for further details.

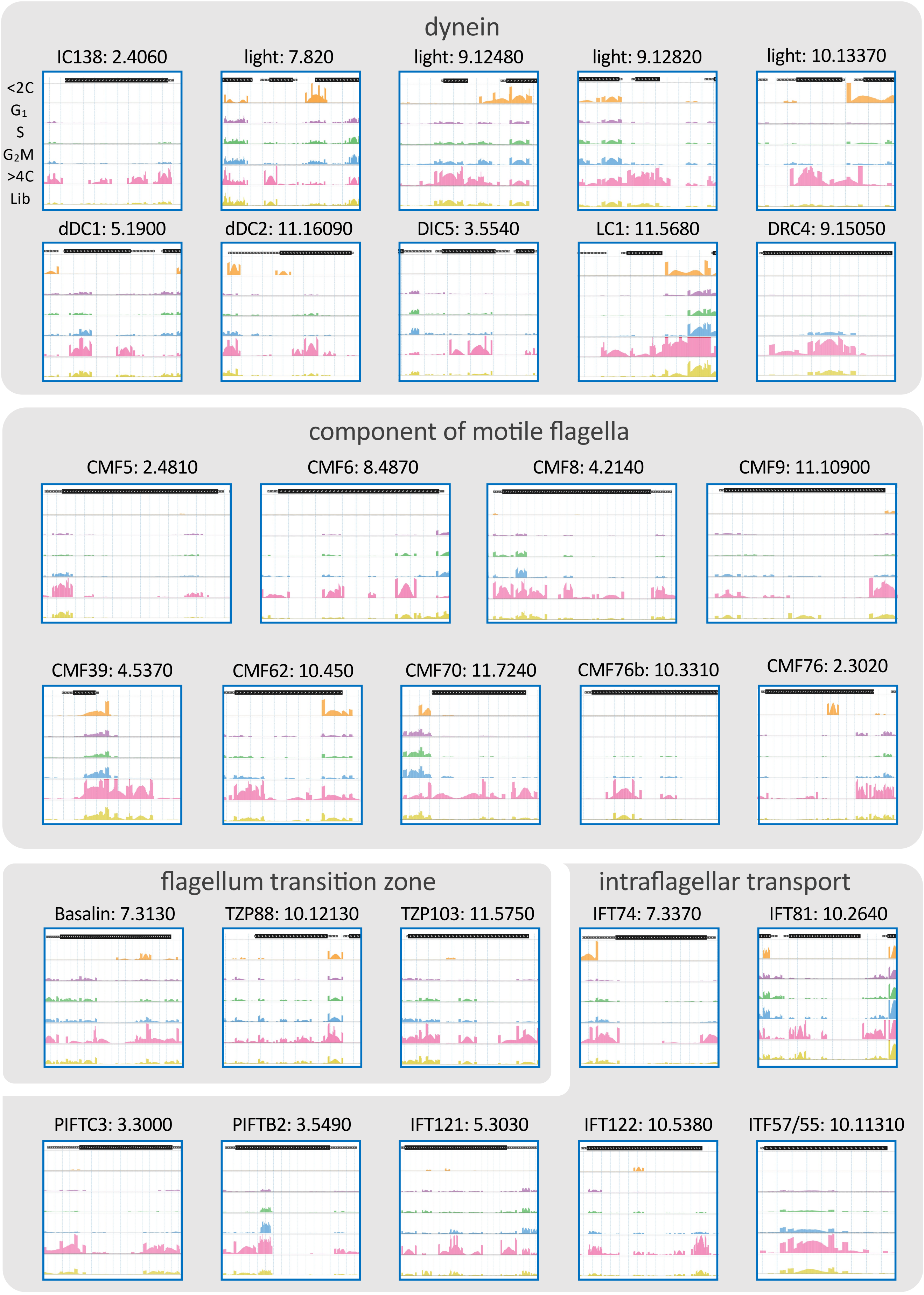

Figure S4 – p2 of 5

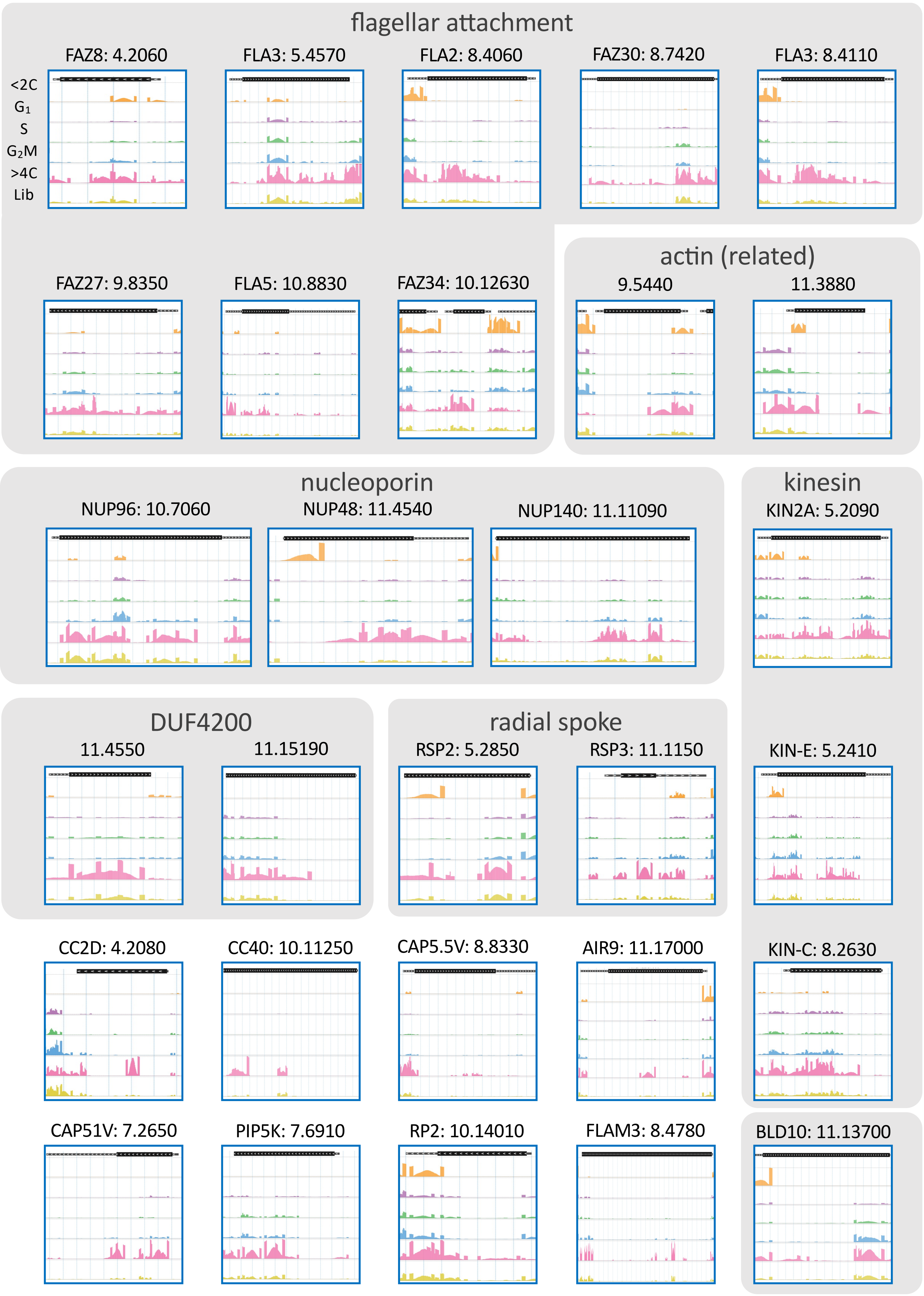

Figure S4– p3 of 5

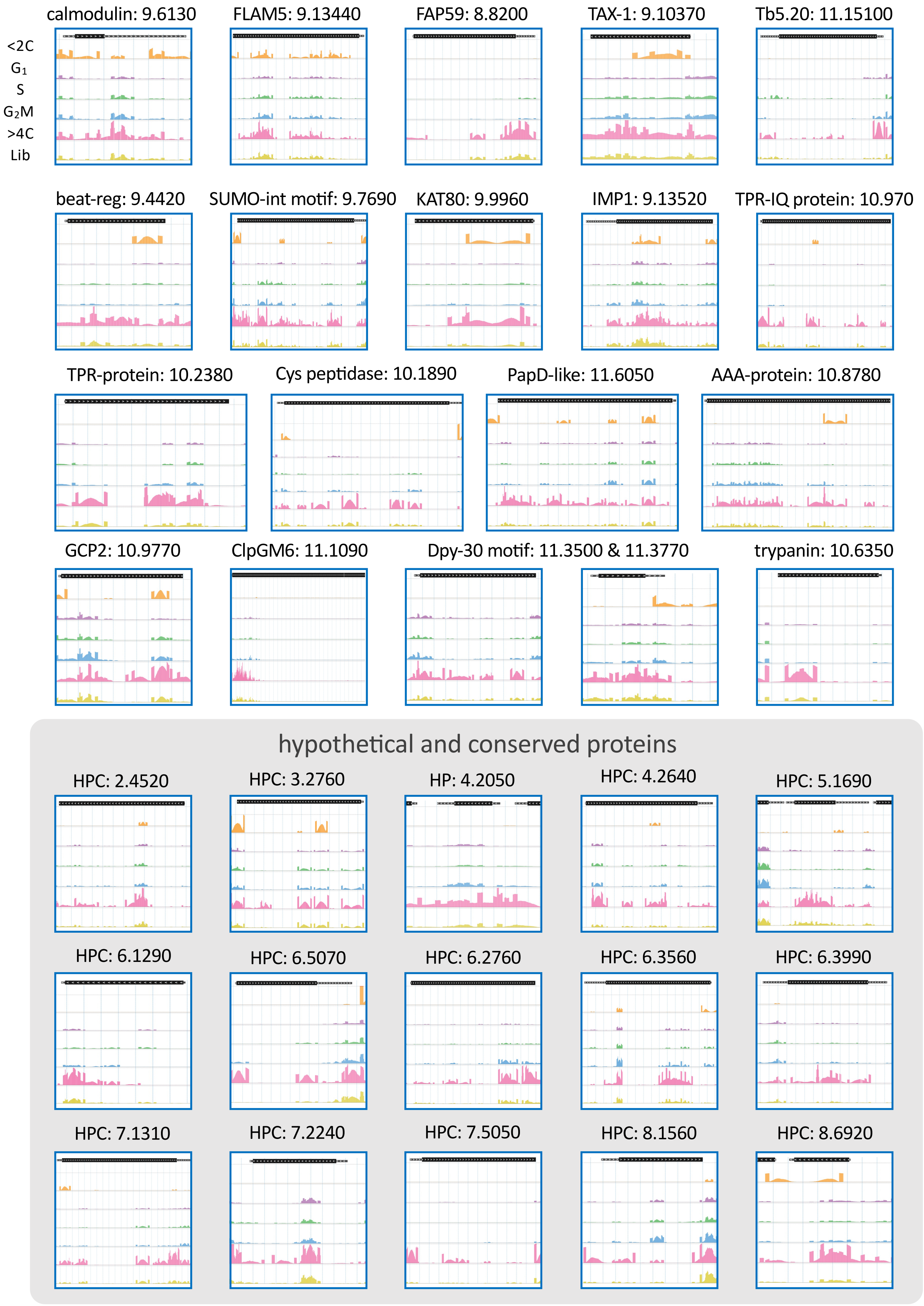

Figure S4 – p4 of 5

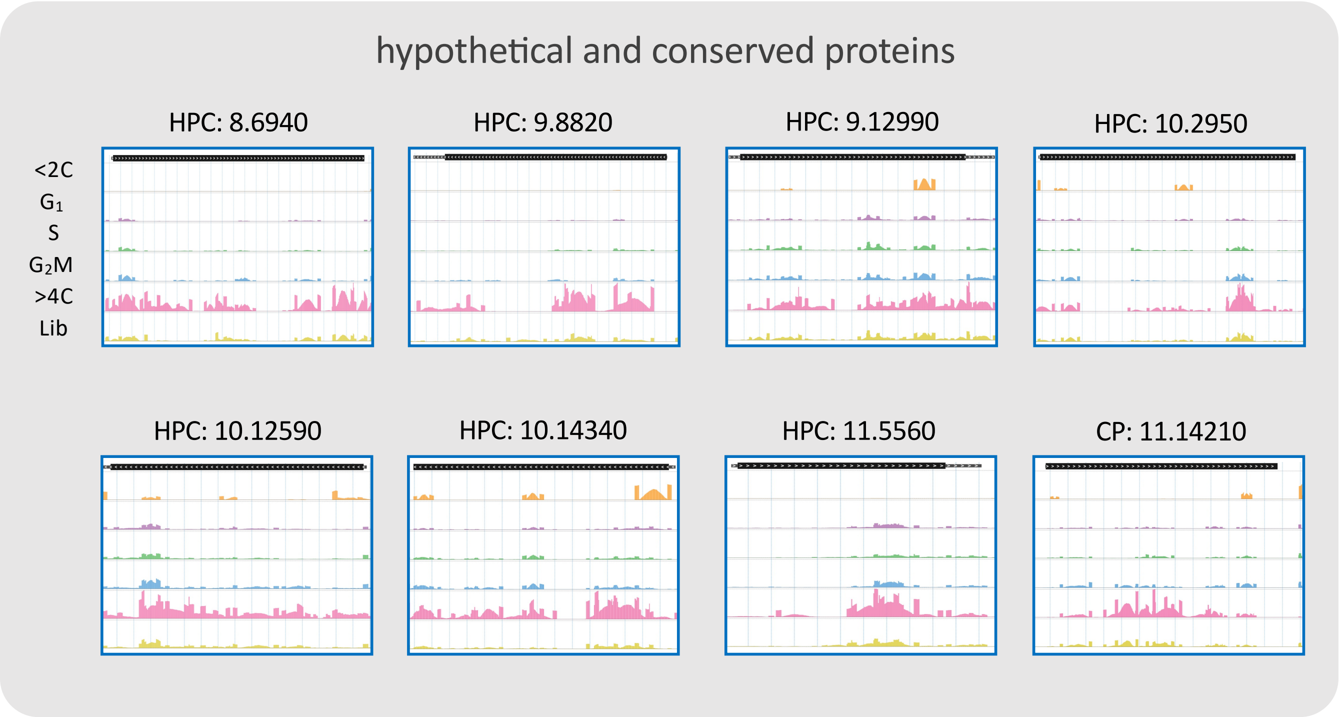

Figure S4 – p5 of 5

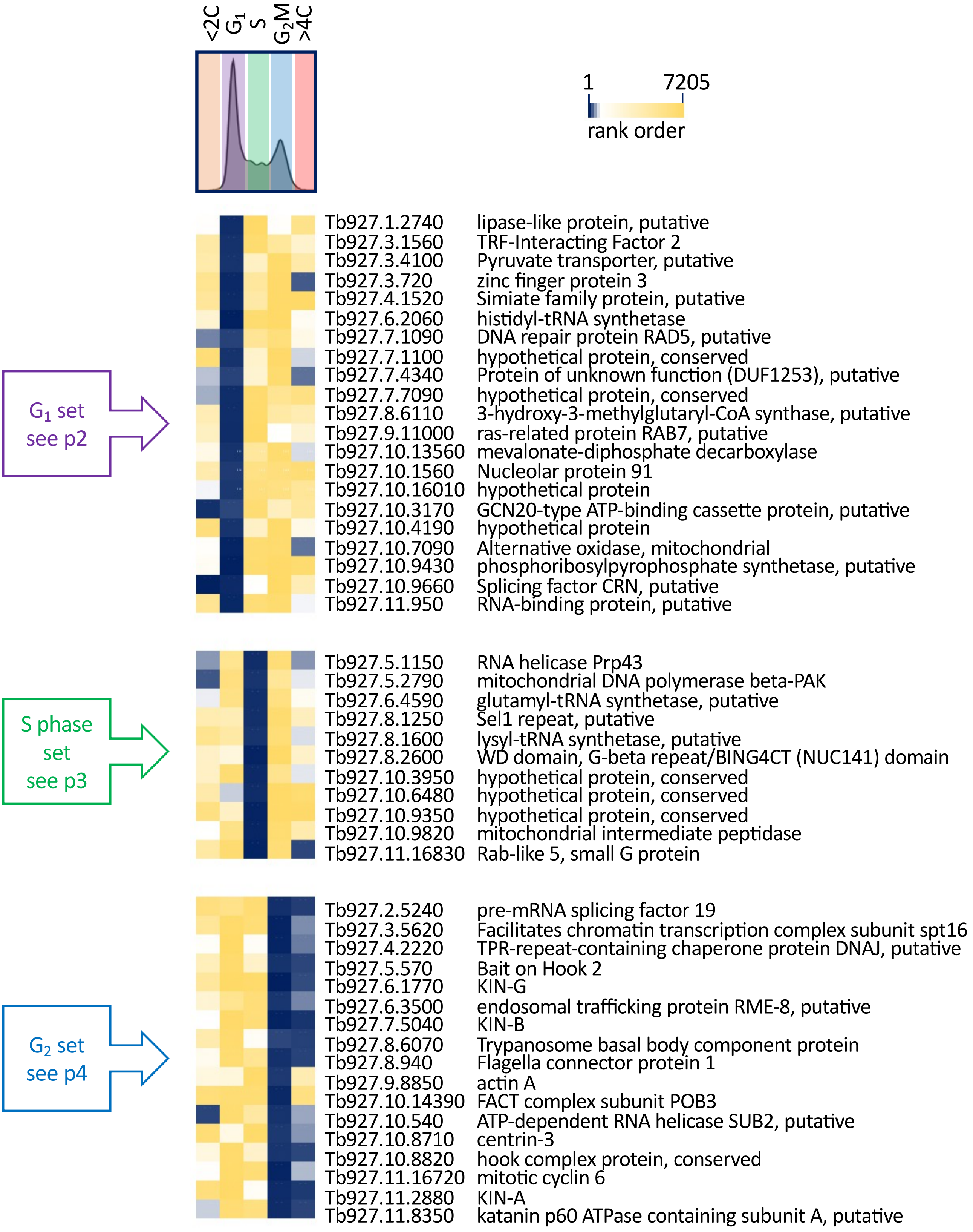

Supplementary Figure 5 Knockdowns associated with gap and S phase defects. Seventy-eight example RIT-seq cell cycle profiles are shown for hits overrepresented in the G_1, S phase and G_2M experiments. Page 1 shows the heatmaps indicating relative representation in all five sorted pools; blue, most overrepresented. Subsequent pages show read-mapping profiles for each gene; see Figure 1b for further details.

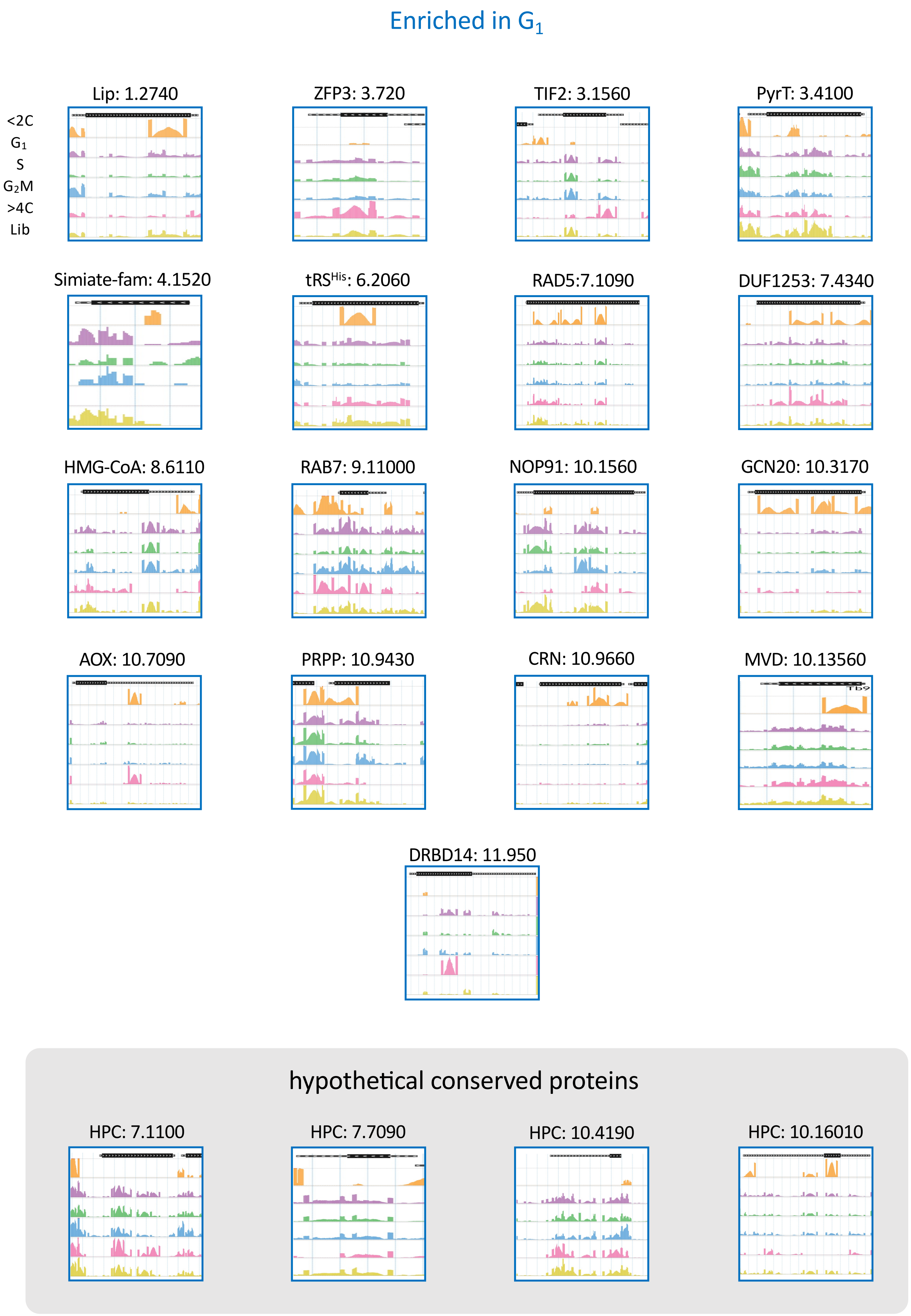

Figure S5 – p2 of 4

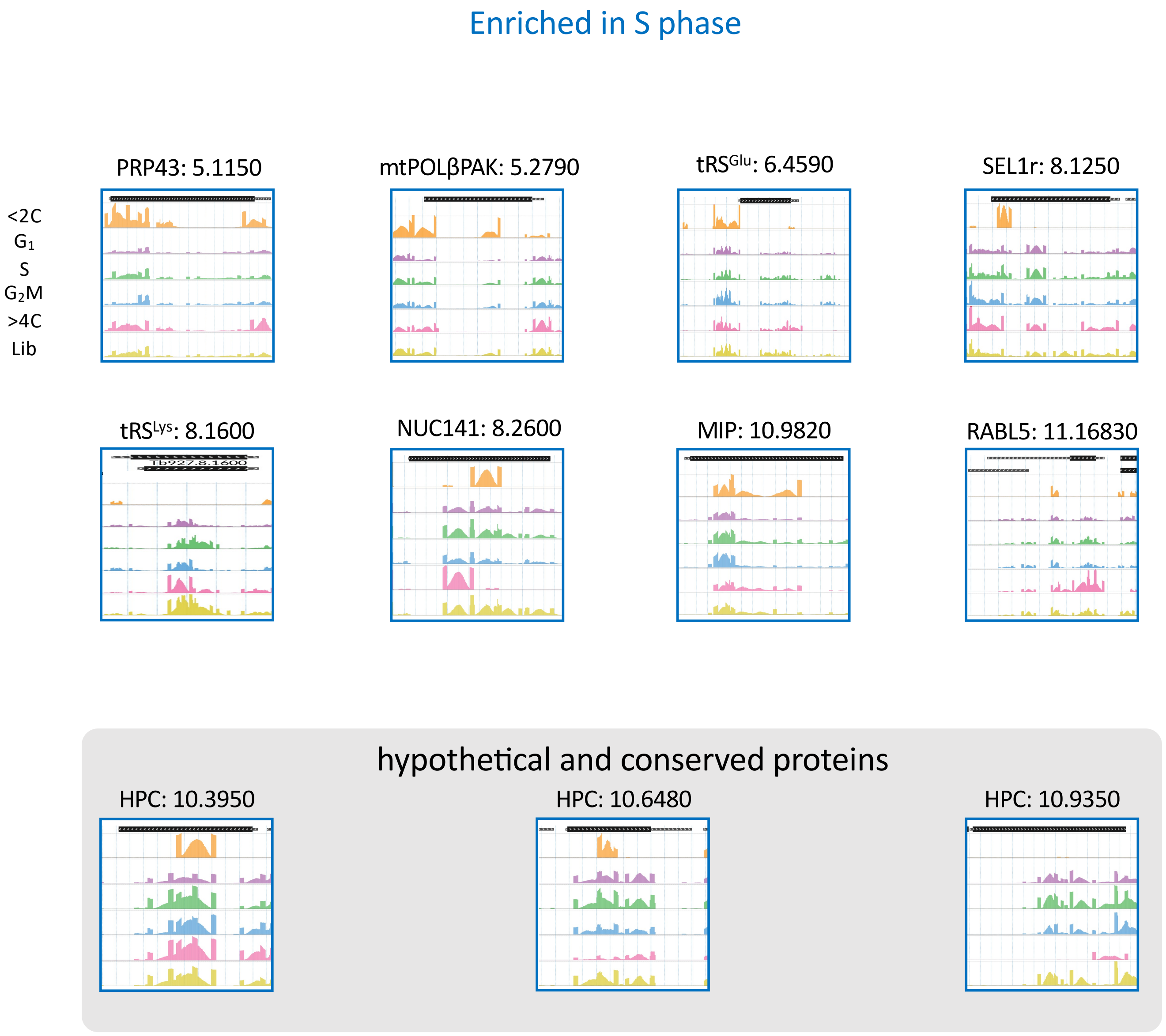
Enriched in S phase
PRP43: 5.1150
mtPOLβPAK: 5.2790
tRSGlu: 6.4590
SEL1r: 8.1250
<2C
G1
S
G2M
>4C
Lib
tRSLys: 8.1600
Tb927.8.1600
NUC141: 8.2600
MIP: 10.9820
RABL5: 11.16830
hypothetical and conserved proteins
HPC: 10.3950
HPC: 10.6480
HPC: 10.9350

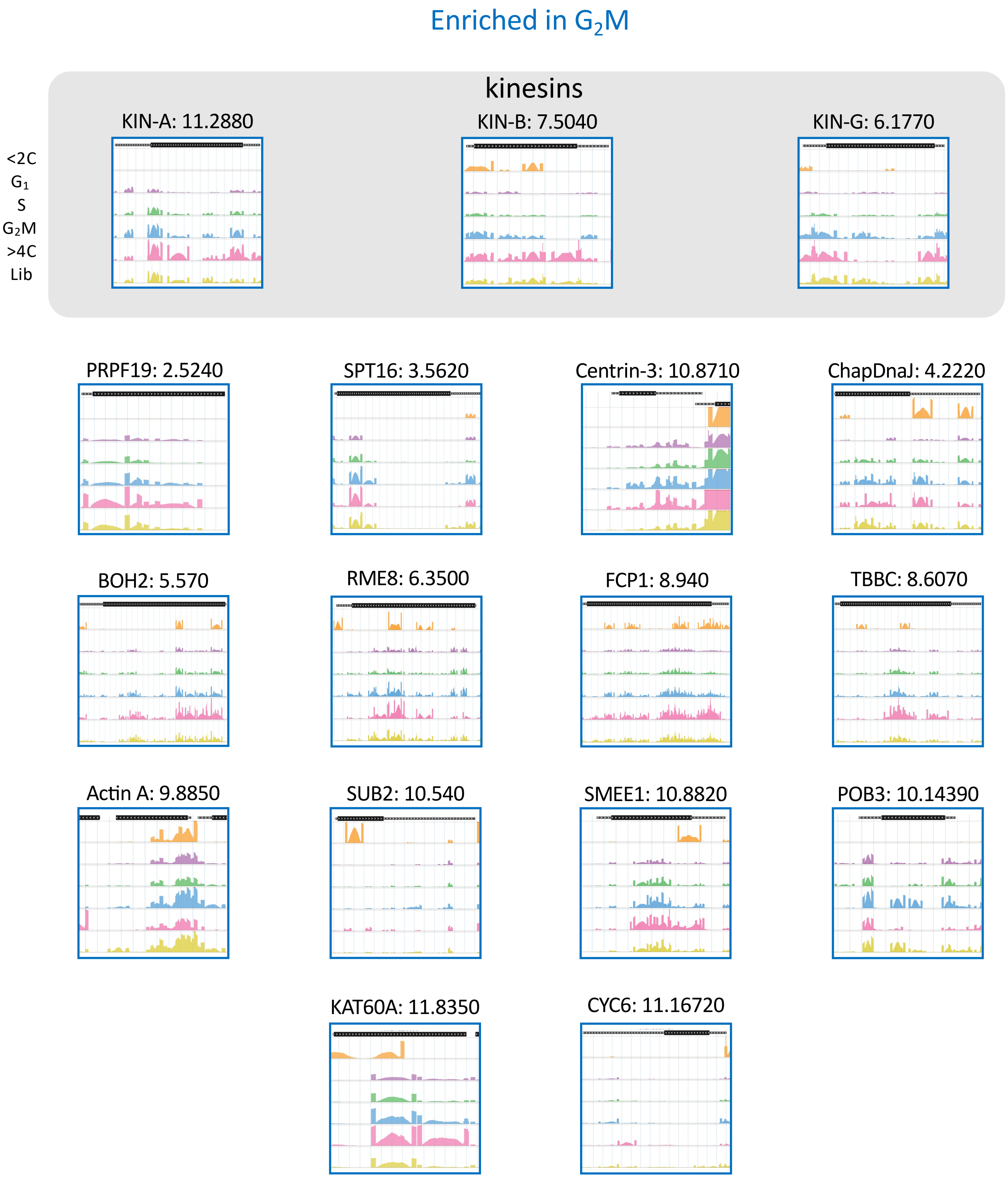

Figure S5 – p4 of 4
