# Supplementary material for: Genome-scale RNA interference profiling of Trypanosoma brucei cell cycle progression defects
Source: Nat Commun. 2022 Sep 10;13:5326. doi: 10.1038/s41467-022-33109-y (PMC9464253; doi:10.1038/s41467-022-33109-y)
Supplement: Supplementary file 3 — Description of Additional Supplementary Information [file 41467_2022_33109_MOESM3_ESM.pdf]

Description of Additional Supplementary Information

Title: Supplementary data 1

Description: Supplementary data 1 RIT-seq digital data. The Excel file reports the total fragment counts for the uninduced (column C) and induced (column D) RNAi libraries and for the five sorted samples (columns E-I), all normalised read-counts for the above (columns J-P), relative enrichment for each sorted sample (columns Q-U) and relative enrichment for barcoded reads for the G1, S phase and G2M sorted samples (columns V-X). Column Y indicates Figure numbers for genes shown in the manuscript. TPM, Transcripts Per kilobase Million. Coloured values in column Q-X indicates ‘hits’, as described in the key and in the figure legends. Data are presented for 7,205 genes, which is 98% of the non-redundant genomic set; all of these genes register >99 total reads across the five sorted samples.
