# Supplementary material for: Genome-scale RNA interference profiling of Trypanosoma brucei cell cycle progression defects
Source: Nat Commun. 2022 Sep 10;13:5326. doi: 10.1038/s41467-022-33109-y (PMC9464253; doi:10.1038/s41467-022-33109-y)
Supplement: Supplementary file 5 — Reporting Summary [file 41467_2022_33109_MOESM5_ESM.pdf]

nature portfolio

Corresponding author(s): David Horn
Last updated by author(s): Aug 22, 2022

Reporting Summary

Nature Portfolio wishes to improve the reproducibility of the work that we publish. This form provides structure for consistency and transparency in reporting. For further information on Nature Portfolio policies, see our Editorial Policies and the Editorial Policy Checklist.

Statistics

For all statistical analyses, confirm that the following items are present in the figure legend, table legend, main text, or Methods section.

n/a	Confirmed	
☐	☒	The exact sample size (*n*) for each experimental group/condition, given as a discrete number and unit of measurement
☐	☒	A statement on whether measurements were taken from distinct samples or whether the same sample was measured repeatedly
☐	☒	The statistical test(s) used AND whether they are one- or two-sided<br>*Only common tests should be described solely by name; describe more complex techniques in the Methods section.*
☒	☐	A description of all covariates tested
☒	☐	A description of any assumptions or corrections, such as tests of normality and adjustment for multiple comparisons
☐	☒	A full description of the statistical parameters including central tendency (e.g. means) or other basic estimates (e.g. regression coefficient) AND variation (e.g. standard deviation) or associated estimates of uncertainty (e.g. confidence intervals)
☐	☒	For null hypothesis testing, the test statistic (e.g. *F*, *t*, *r*) with confidence intervals, effect sizes, degrees of freedom and *P* value noted<br>*Give P values as exact values whenever suitable.*
☒	☐	For Bayesian analysis, information on the choice of priors and Markov chain Monte Carlo settings
☒	☐	For hierarchical and complex designs, identification of the appropriate level for tests and full reporting of outcomes
☒	☐	Estimates of effect sizes (e.g. Cohen's *d*, Pearson's *r*), indicating how they were calculated

Our web collection on statistics for biologists contains articles on many of the points above.

Software and code

Policy information about availability of computer code

Data collection	For RIT-seq: base call, index deconvolution, trimming and QC were performed in BaseSpace using bcl2fastq2 Conversion Software v2.17.
Data analysis	Bowtie2, SAMtools, Qualimap 2, MultiQC, Picard tools, Samtools to analyse the fastq files.<br>radvizs, vist4get and the SciPy Python packages for the analysis of the read counts.<br>bash script containing the analysis pipeline, a conda environment specification file for its execution, the python script to extract barcoded reads and and analyse the read counts are available at GitHub (https://github.com/mtinti/ritseq_cellcycle).<br>The software package versions used for the analysis are listed in the file ritseq.yml file (DOI: 10.5281/zenodo.7002689).<br>FlowJo 10.7.1, ImageJ v1.53q.

For manuscripts utilizing custom algorithms or software that are central to the research but not yet described in published literature, software must be made available to editors and reviewers. We strongly encourage code deposition in a community repository (e.g. GitHub). See the Nature Portfolio guidelines for submitting code & software for further information.

Data

Policy information about availability of data

All manuscripts must include a data availability statement. This statement should provide the following information, where applicable:
- Accession codes, unique identifiers, or web links for publicly available datasets
- A description of any restrictions on data availability
- For clinical datasets or third party data, please ensure that the statement adheres to our policy

The high-throughput sequencing data generated in this study have been deposited in the Short Read Archive (SRA) under accession code PRJNA641153 [https://www.ncbi.nlm.nih.gov/sra/PRJNA641153]. The mapped data can be visualised using an online tool at https://tryp-cycle.pages.dev/. Source data are provided with

March 2021

this paper for Figures 7c, 8c and 8e-g. Other data relating to individual genes can be found at tritrypdb.org.

Field-specific reporting

Please select the one below that is the best fit for your research. If you are not sure, read the appropriate sections before making your selection.

☒ Life sciences ☐ Behavioural & social sciences ☐ Ecological, evolutionary & environmental sciences

For a reference copy of the document with all sections, see nature.com/documents/nr-reporting-summary-flat.pdf

Life sciences study design

All studies must disclose on these points even when the disclosure is negative.

Sample size	We used two biological replicates for RNAi analysis of Tb927.10.970 or Tb927.10.3970, including analysis on growth following knockdown, flow cytometry, protein blotting and microscopy. When counting DAPI-stained cells, n=100 is typical and desirable. When quantifying DNA-intensity or GFP-intensity in DAPI-stained cellular compartments, n=50-100 is considered sufficient.
Data exclusions	No data were excluded from the analyses.
Replication	Two of us observed the trends reported in Figures 8e-g using a series of preliminary experiments. Three technical replicates are considered to provide sufficiently robust qRT-PCR data. All attempts at replication were successful.
Randomization	We use a strain with RNAi cassettes expressed from a locus that has been validated for robust and reproducible, inducible knockdown. Accordingly, two clones that behave similarly are considered sufficient for cross-validation and randomization is not considered necessary..
Blinding	Blinding was not considered necessary since the ImageJ and flow cytometry assays were automated. In addition, the perturbed cells analyzed here were easily distinguished from unperturbed cells using the microscopy-based assays.

Reporting for specific materials, systems and methods

We require information from authors about some types of materials, experimental systems and methods used in many studies. Here, indicate whether each material, system or method listed is relevant to your study. If you are not sure if a list item applies to your research, read the appropriate section before selecting a response.

Materials & experimental systems

n/a	Involved in the study	
☐	☒	Antibodies
☐	☒	Eukaryotic cell lines
☒	☐	Palaeontology and archaeology
☒	☐	Animals and other organisms
☒	☐	Human research participants
☒	☐	Clinical data
☒	☐	Dual use research of concern

Methods

n/a	Involved in the study	
☒	☐	ChIP-seq
☐	☒	Flow cytometry
☒	☐	MRI-based neuroimaging

Antibodies

Antibodies used	ANTIBODY / SOURCE / IDENTIFIER<br>Mouse anti-Myc 9B11 / Cell Signaling Technology/ Cat# 2276S RRID:AB_331783<br>Rabbit anti-GFP / Abcam / Cat# 290 RRID:AB_303395<br>Mouse anti-EF1α CBP-KK1 / Merck-Millipore / Cat# 05-235 RRID:AB_309663<br>Goat anti-mouse HRP / Biorad / Cat# 1721011 RRID:AB_11125936<br>Goat anti-rabbit Alexa 488 / ThermoFisher Scientific / Cat# A-11008 RRID:AB_10563748
Validation	All antibodies have been widely used and validated for the purposed reported here.

Eukaryotic cell lines

Policy information about cell lines

Cell line source(s)	Trypanosoma brucei brucei Lister 427 was originally obtained from Prof. George Cross (Rockefeller University, NYC, USA). Subsequent genetic modifications were performed by the authors.
Authentication	RIT-seq and microscopy provided authentication.

Mycoplasma contamination	Mycoplasma contamination check carried our approx. every 3 years - no positive results from those tests to date.
Commonly misidentified lines (See ICLAC register)	T. b. brucei L427 is not a commonly misidentified line.

Flow Cytometry

Plots

Confirm that:

- [x] The axis labels state the marker and fluorochrome used (e.g. CD4-FITC).
- [x] The axis scales are clearly visible. Include numbers along axes only for bottom left plot of group (a 'group' is an analysis of identical markers).
- [x] All plots are contour plots with outliers or pseudocolor plots.
- [x] A numerical value for number of cells or percentage (with statistics) is provided.

Methodology

Sample preparation	Cells were fixed with 1% formaldehyde, permeabilised with Triton X-100, treated with RNaseA and stained with propidium iodide (PI).
Instrument	BD InfluxTM (Becton Dickinson) - RIT-seq screen.<br>FACS LSR Fortessa flow cytometry analyser - post-sorting quality check.<br>BD FACSCanto (Becton Dickinson) - 10.970 / 10.3970 analysis.
Software	BD FACSortTM.<br>FlowJo v10.
Cell population abundance	~50 M cells were sorted for the RIT-seq screen.<br>>1000 cells were analysed for the post-sorting quality check and for 10.970 / 10.3970 analysis.
Gating strategy	The cells were gated using the PI staining - see Figure 7d, Figure 8d and Supplementary Figure 1A. The gates for each cell cycle stage were defined based on the profile of uninduced (- tet) cells.

- [x] Tick this box to confirm that a figure exemplifying the gating strategy is provided in the Supplementary Information.
